# Exploring the impact of stigma on the health of inclusion health groups: a qualitative scoping review and critical analysis

**DOI:** 10.1186/s12889-025-25603-x

**Published:** 2025-11-24

**Authors:** Timothy Price, Victoria McGowan, Christina Cooper, Steph Scott

**Affiliations:** 1 Faculty of Medical Sciences, Population Health Sciences Institute, Newcastle University, Newcastle Upon Tyne, NE1 4LP UK; 2 Department of Social Work, Education and Community Wellbeing, Faculty of Health and Life Sciences, Northumbria University, Newcastle Upon Tyne, NE7 7XA UK

**Keywords:** Stigma, Inclusion health, Scoping review, Qualitative

## Abstract

**Background:**

Health inequalities have widened globally over the past decade, disproportionately affecting socially excluded populations broadly defined as ‘inclusion health groups’. These groups, including people experiencing homelessness, migrants, sex workers, people with substance use disorders, victims of modern slavery, and those in contact with the justice system, face compounded negative health consequences often exacerbated by stigma. This scoping review aimed to examine qualitative literature exploring how stigma impacts the physical and mental health of inclusion health groups. Building on the work of Link and Phelan, Tyler, and Hatzenbuehler, this review situates stigma as a relational and structural process that operates through power, policy, and institutions to shape health outcomes among inclusion health populations.

**Methods:**

The review focused on identifying how stigma is conceptualised across inclusion health groups and how it functions as a shared mechanism influencing health, health behaviour, and access to care. Following the PRISMA-ScR framework, we searched Scopus, OVID Medline, and PsycINFO for qualitative studies published between 01/01/2015 and 15/03/2025. Titles, abstracts, and full texts were screened, resulting in 28 articles included for analysis.

**Results:**

Stigma was consistently identified as a barrier to healthcare access, leading to delayed treatment and worsening physical and mental health outcomes for inclusion health groups. The literature disproportionately focuses on people who use drugs, with limited research addressing other inclusion health groups, highlighting significant gaps in the field. Furthermore, existing conceptualisations of stigma frequently neglect its structural determinants, risking reinforcement of individualised explanations for poor health rather than addressing systemic drivers of inequality.

**Conclusions:**

This review demonstrates that stigma contributes to health inequalities by limiting healthcare access and shaping negative health outcomes. There is an urgent need for research that investigates stigma’s long-term health effects and moves beyond individual-level interventions to address broader structural forces perpetuating health inequalities. Future work should more explicitly engage with the concept of structural and political stigma, recognising that public health research must interrogate the upstream determinants, such as policy, governance, and social organisation, that sustain exclusion and health inequity.

**Supplementary Information:**

The online version contains supplementary material available at 10.1186/s12889-025-25603-x.

## Background

Over the past decade, health inequalities have widened globally. In both the UK and the USA, life expectancy among the most disadvantaged populations has declined, while more privileged groups have disproportionately benefited from improvements in health and wellbeing [1–3]. Across Europe, mortality trends reveal a growing disparity, highlighting how systemic inequalities continue to shape health outcomes [4, 5]. These widening health gaps have been further exacerbated by multiple crises, including the COVID-19 pandemic and rapid increases in cost of living, which amplified social, economic, political, and cultural inequities [6–8].

‘Inclusion health’ is a term that refers to individuals and communities who are socially excluded, experience multiple overlapping health risks (e.g., poverty, violence, trauma), and face significant barriers to accessing healthcare [9]. NHS England defines inclusion health groups as those who are marginalised due to their social status and disproportionately impacted by stigma and discrimination. Inclusion health groups include people experiencing homelessness, individuals in contact with the criminal justice system, migrant populations, Gypsy, Roma, and Traveller communities, sex workers, people with substance use disorders, and others facing severe multiple disadvantage [10, 11]. These groups are not only more vulnerable to poor health outcomes but are also frequently missing from, or underrepresented, in health data further entrenching their exclusion [9]. While the Inclusion Health framework acknowledges that these groups experience stigma and discrimination, this recognition often remains descriptive rather than analytical. In practice, policy and research within Inclusion Health tend to overlook the structural and intersectional dimensions of stigma, focusing instead on individual vulnerability rather than the wider systems that produce exclusion [12]. As such, the focus remains on the individuals themselves rather than the underlying structural factors that intensify stigma and significantly influence their health and wellbeing [12, 13].

Stigma plays a critical role in reinforcing and deepening the health inequalities experienced by inclusion health groups [14, 15]. The concept of stigma remains contested and subject to debate [11, 16].The contested nature of stigma reflects ongoing debates about whether it should be understood primarily as an individual attribute, a social process, or a structural mechanism of inequality. Some scholars argue that the term risks oversimplifying complex social relations or obscuring the political and economic forces that produce marginalisation [17, 18]. However, we retain the concept of stigma in this review because it continues to offer a valuable lens for understanding how power, exclusion, and inequality are embodied and experienced. Stigma provides a way of connecting lived experience to broader social structures, making it a useful analytical framework for examining how social disadvantage becomes translated into health inequality.

The foundational understanding of stigma in sociological research stems from Erving Goffman’s work, which defines stigma as ‘an attribute that is deeply discrediting’ (1968:13) and emphasises its relational nature. However, while Goffman’s framework has been influential, scholars such as Scambler [19] and Tyler [18] argue that it overlooks how stigma is embedded within broader interconnected systems of capitalism, power, and governmentality. Building on these perspectives, Link and Phelan’s [20] conceptualisation of stigma offers a useful framework for understanding how stigma operates through interconnected processes of labelling, stereotyping, separation, status loss, and discrimination, all of which are contingent upon existing power relations [20]. Their later work extends this analysis through the concept of stigma power, which emphasises how stigma functions as a tool of social control, reinforcing existing hierarchies and sustaining structural inequalities [21]. Tyler’s [18] has advanced this argument by situating stigma within political and economic systems, framing it as a mechanism through which inequality is materially produced and maintained. In this review, our conceptualisation of stigma is informed by this body of work, recognising both its structural and health-related dimensions. In particular, Hatzenbuehler’s [22] framing of stigma as a fundamental cause of population health inequalities highlights how stigma restricts access to power, resources, and social capital essential for health, thereby linking social exclusion directly to health inequity. We therefore conceptualise stigma as a relational and structural process that operates through interactions between individuals and institutions, shaping access to health and care while reproducing broader social and political inequalities.

Several prior reviews have used mixed methods to examine stigma and health within specific populations included under the inclusion health framework. For example, Yang et al. [23] reviewed stigma, violence, and coping among transgender people engaged in sex work,Cabieses et al. [24] reported on stigma and discrimination in relation to migrant health in Latin America, Douglas et al. [25] explored stigma associated with alcohol and other drug use among migrant and ethnic minority groups; and a review by Martin et al. [26] examined how criminal justice stigma affects health and healthcare utilisation. These reviews provide valuable insights into how stigma operates within particular inclusion health groups and contexts. However, they focus on single populations or specific forms of stigma, often without situating findings within the broader structural conditions that link experiences of stigma across inclusion health groups and have drawn on mixed methods. Our review compliments this literature by synthesising qualitative evidence across multiple inclusion health populations to identify common mechanisms through which stigma shapes physical and mental health, and by critically examining how these mechanisms reflect and reproduce wider social and structural inequalities. To our knowledge, no published review has identified and synthesised qualitative studies exploring the impact of stigma on the health and wellbeing of inclusion health groups more broadly. Understanding what is already known about how stigma intersects with structural disadvantage to impact health and wellbeing is an essential step towards identifying evidence gaps and formulating research questions designed to address the broader social determinants of health inequalities and improve health outcomes for those most excluded from care.

## Methods

### Review design

We conducted a scoping literature review to explore and map the existing qualitative literature on the impact of stigma on the health and wellbeing of individuals belonging to inclusion health groups [27]. A review protocol was registered online via the Open Science Framework (Registration 10.17605/OSF.IO/4VGDH) [28, 29]. This review was conducted in-line with the Preferred Reporting Items for Systematic reviews and Meta-Analyses extension for Scoping Reviews (PRISMA-ScR) tool [30]. This review had two specific research questions:

- How do inclusion health groups experience stigma?
- What impact does stigma have on the physical and mental health and wellbeing of inclusion health groups?

### Search Strategy and inclusion/exclusion criteria

We searched three online bibliographic databases (Scopus, Medline, and PsychINFO) for literature published between 01/01/2015 and 15/03/2025. These databases were chosen to ensure comprehensive coverage, as they include those relevant to healthcare research (PsychINFO, Medline) and a broad multidisciplinary database (Scopus). Additionally, they are hosted by separate providers, EBSCOhost (Scopus), ProQuest (PsychINFO), and OVID (Medline), which further diversifies the sources of indexed literature. Together, these choices were intended to maximise the breadth and capture of relevant research while maintaining a streamlined review process compatible with the scoping review design.

We chose to include only studies that used a qualitative study design, to gain a deeper understanding of the lived experiences of stigma and its impact on the health of inclusion health groups. While inclusion health is a broad term that can encompass a wide range of populations, it was beyond the scope of this review to include all conceivable inclusion health groups. Therefore, we restricted inclusion to groups defined in NHS England’s Inclusion Health framework [31]. Further inclusion and exclusion criteria are shown in Table 1 below; the search strategy for this review is included as supplementary material to this article (see Supplementary Table 01).

**Table 1:** Inclusion and exclusion criteria

Inclusion:	Exclusion:
Studies reporting primary data of any qualitative design, for example: ethnographic studies, studies that used a phenomenological or grounded theory approach, or participatory action research	Unpublished data, abstracts, conference proceedings and studies including no primary data (e.g. protocols, editorials, reviews)
Studies published in English language from 2015 onwards, originating from OECD countries. Date and country limits are designed to ensure included studies are comparable to the context of the United Kingdom and to minimise outdated findings by focusing on data collected post-austerity measures	Studies that use self-report or researcher-administered surveys, including those which attempt to analyse data from open-ended questions, as the sole method of data collection, as it is felt that survey data cannot explore the topic in sufficient depth Qualitative research that has not ascertained lay perspectives but has analysed texts e.g. discourse analysis
Studies that explore the impact of stigma on the physical and mental health and wellbeing of inclusion health groups as specifically outlined by NHS England (people experiencing homelessness, individuals in contact with the criminal justice system, migrant populations, people in indentured servitude and modern slavery, Gypsy, Roma, and Traveller communities, sex workers, people with substance use disorders)	Place-based studies
Studies where participants are defined as adults (over the age of 18). Studies that focus retrospectively on experiences that adult participants had in childhood also meet inclusion criteria for this review, for example: experiences of care, ACEs, looked-after-children	Participants under the age of 18

Date restrictions were applied to ensure temporal relevance to current political and socioeconomic circumstances. Further, the term ‘stigma’ is both experienced and perceived cross-culturally. Therefore, restricting to high-income countries only allows a more nuanced understanding within this cultural context.

### Study selection, screening, and data extraction

**Fig. 1:**
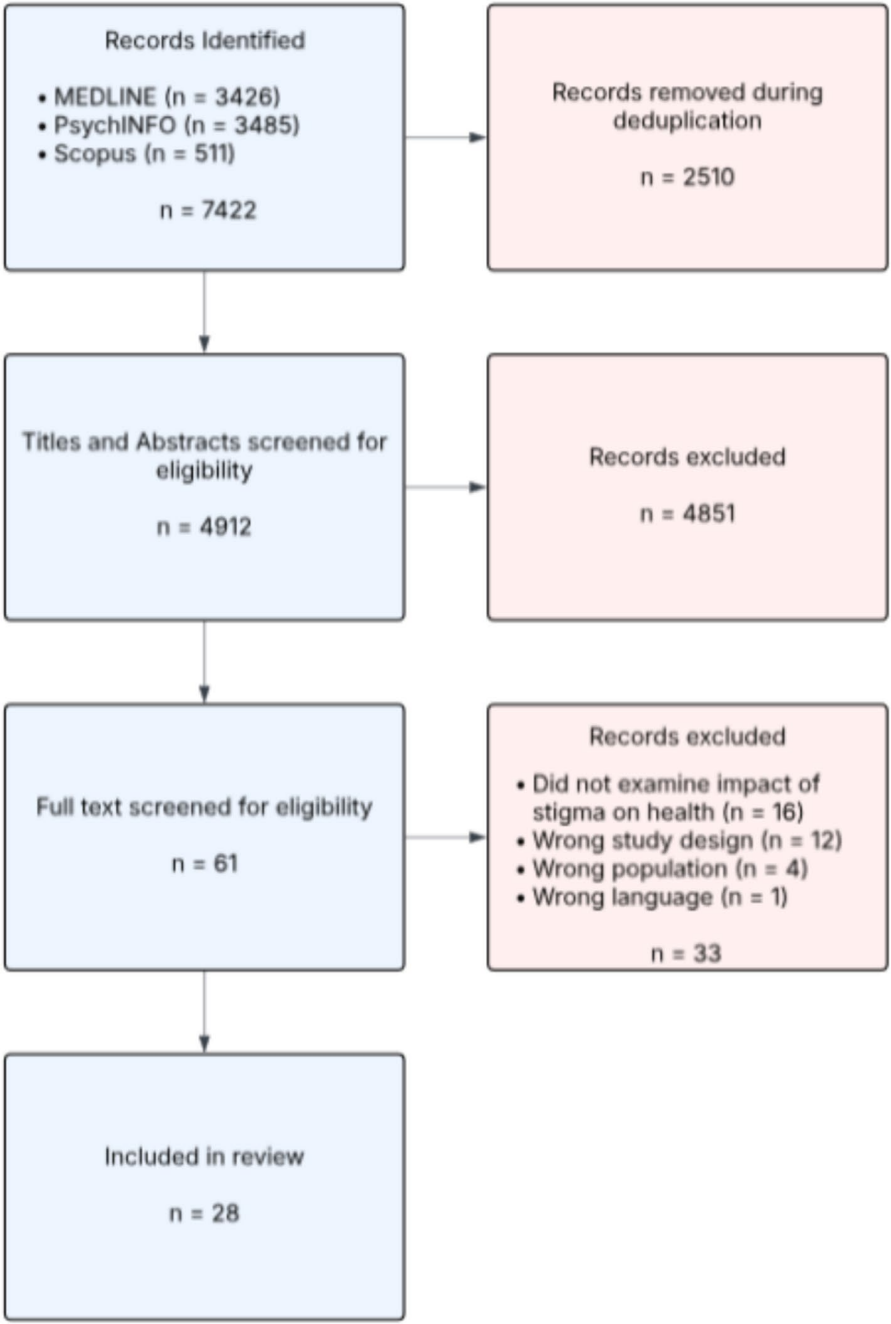
Review flow chart

The primary reviewer (TJP) ran the initial searches across all online databases to identify empirical articles in the field. The title and abstract of all records retrieved were uploaded to Rayyan (https://rayyan.qcri.org/welcome) and duplicates were removed. The title and abstract screening process was carried out by a single reviewer (TJP) for the majority of articles. However, to ensure a high degree of agreement on the inclusion of articles, 20% of the records underwent double screening, with both reviewers (TJP and VJM) independently assessing these records. This subset served to verify the consistency and reliability of the screening process and was repeated for full text screening (TJP and VJM) and data extraction (TJP and CC). At each stage, a high degree of concordance was achieved between the two reviewers. Reasons for exclusion were noted at the full text stage. Figure 1 presents a flow chart of the study selection process. Data extraction was conducted using a customised data extraction form. Information extracted using the study form included: Authors, year of publication, study setting, study design, participant details, method of analysis, and findings. In line with guidance on scoping review conduct, we did not carry out a quality appraisal or risk of bias assessment of the included articles [32].

### Data analysis

We employed a narrative approach to the synthesis of the extracted data surrounding the impact of stigma on the health and wellbeing of inclusion health groups. The initial coding and theme generation were carried out primarily by one author (TJP), who closely reviewed all data extracted from included studies and identified recurrent patterns and concepts. A second author (VJM) independently verified the extracted data and initial themes to ensure consistency, accuracy, and completeness. Throughout the analysis, the research team engaged in reflexive discussions to critically evaluate the emerging themes, resolve discrepancies, and refine the synthesis. These discussions helped to ensure that the themes were coherent, representative of the underlying evidence, and adequately captured nuances across different study populations and settings. Themes were iteratively revised as new insights emerged, resulting in a comprehensive and structured narrative synthesis.

## Results

### Characteristics of included studies

After full text screening, twenty-eight studies were included for data extraction. Included studies collected data from four inclusion health groups: people who use drugs (*n* = 21; 75%), migrants (*n* = 5; 18%), Indigenous people (*n* = 1; 3.8%) and sex workers (*n* = 1; 3.8%). No studies were identified that examined the impact of stigma on the health of people in contact with the criminal justice system, people who are homeless, people in indentured servitude and modern slavery, or members of Gypsy, Roma, and Traveller communities. The included studies included populations in the USA (*n* = 14; 50%), Canda (*n* = 9; 32%), Belgium (*n* = 1; 3.8%), the UK (*n* = 2; 7.6%), Germany (*n* = 1; 3.8%), and multiple European countries (*n* = 1; 3.8%). The methodology used to generate the qualitative data included interviews (*n* = 21; 75%), focus groups (*n* = 5; 18%), talking circles (*n* = 1; 3.8%), or a combination of interviews and participant observation (*n* = 1; 3.8%). Findings were generated using thematic analysis (*n* = 17; 61%), content analysis (*n* = 4; 14.3%), or framework analysis (*n* = 4; 14.3%). Three studies (11%) did not specify their analytic methods.

The findings of this review are presented thematically. Organising the findings thematically rather than by population allows for a deeper exploration of the common ways stigma influences health and well-being across different inclusion health groups. This approach highlights overarching patterns and mechanisms through which stigma operates, rather than segmenting experiences by group, which may obscure shared themes and broader implications. Table 2 presents the included studies.

**Table 2:** Characteristics of included studies

Study	Location	Inclusion health group	Overlapping Stigma*	Sample size	Data collection and analytic methods	Principle findings
[33]	Belgium	Sub-Saharan migrant women	HIV Infection	44	Participant observation and interviews. Thematic analysis	Stigma resulted in non-disclosure of HIV status to healthcare providers, low self-esteem and depression, and avoidance of seeking healthcare
[34]	USA	People who use drugs who live with HIV	HIV Infection	33	Semi-structured interviews. Thematic analysis	Internalized stigma surrounding drug use led to further drug use and avoidance of healthcare services
[35]	USA	People who are in recovery for opioid use disorder	N/A	58	Focus groups. Summative content analysis	Stigma surrounding opioid use lead people to avoid accessing recovery support services
[36]	Canada	People who use drugs who live with HIV	HIV Infection	24	Interviews. Thematic analysis with a realist perspective	Stigma impacted the quality of care received when engaging health services. Negative experiences with healthcare providers lead to care avoidance in the future
[37]	USA	People who use drugs	N/A	24	Semi-structured interviews. Analytic method not specified	Healthcare providers took health concerns less seriously because of drug use stigma. These experiences lead people who used drugs to avoid accessing care in the future
[38]	Canada	Recent migrants to Canada	Mental Health	10	Interviews. Thematic analysis	Immigrants managed their mental health without professional support because of stigma surrounding mental health treatment in some immigrant communities
[39]	USA	Women accessing medication for opioid use disorder	N/A	20	Interviews. Thematic analysis informed by constructivist grounded theory	Internalised stigma impedes peer support and treatment engagement for women in recovery
[40]	Canada	People who use drugs	Hepatitis-C Infection	15	Semi-structured interviews. Framework analysis using theoretical domains framework	Stigma led to judgment and discrimination from healthcare providers, leading to healthcare avoidance
[41]	Canada	People who use drugs	N/A	114	Interviews. Thematic ethnographic analysis	Lack of trust due to stigmatising experiences led to reduced help-seeking and healthcare engagement. Lack of engagement led to infections, unsafe needle use, discharge against medical advice, and risk of death
[42]	Canada	People who use drugs	N/A	104	Interviews. Thematic ethnographic analysis	Stigma surrounding methamphetamine use led to unfair treatment and unmet medical needs among people who use drugs, leading to avoidance of future care and poor health outcomes
[43]	Canada	People who use drugs	N/A	19	Semi-structured interviews. Thematic analysis	People who use drugs felt stigmatised and discriminated against when accessing emergency care, leading to care avoidance
[44]	USA	People who use drugs	N/A	16	Interviews. Thematic analysis	Opioid use was considered shameful leading to people avoiding treatment to avoid stigmatisation
[45]	Canada	Indigenous people	N/A	35	Talking circles. Multi-phased analysis	Stigmatising narratives surrounding "drug seeking natives" led to mistreatment when accessing healthcare. Stigmatising experiences led to future healthcare avoidance
[46]	Canada	People who use drugs	Hepatitis-C Infection	50	Interviews. Thematic analysis	Stigma deterred people from accessing harm reduction services, leading to more dangerous drug use practices (e.g. reusing needles, using alone)
[47]	USA	People who use drugs	N/A	28	Interviews. Thematic analysis	Judgement from family members, friends, and healthcare providers deterred access to medication supported sobriety
[48]	USA	People who use drugs	N/A	41	Semi-structured interviews. Thematic analysis informed by constructivist grounded theory	Anticipated stigma prevented people from using syringe exchange programmes
[49]	USA	People who use drugs	N/A	30	Semi-structured interviews. Thematic analysis	Stigmatising views of drug use led people to conceal their drug use, delay access to care and harm reduction services, and created feelings of low-mood and shame
[50]	UK	Mothers who use drugs	N/A	13	Focus groups. Reflexive thematic analysis	Previous stigmatising experiences led people to avoid seeking support for their substance use and resulted in poor mental health and low self-esteem. Stigma from providers also led to mothers who use drugs being deemed "too complex" to support in routine services
[51]	USA	People who use drugs	N/A	10	Focus groups. Analytic method not specified	Participants refused medication to assist sobriety due to fear of being labelled "an addict". Stigma against people who use drugs from healthcare providers led to poor quality care, and stigma prevented them from accessing services in the future
[52]	USA	People who use drugs	N/A	32	Semi-structured interviews. Thematic analysis using grounded theory	Stigma from healthcare providers caused people to withdraw from care early and avoid care in the future
[53]	USA	People who use drugs	N/A	46	Interviews. Framework analysis	Participants reported they avoided healthcare and support services due to past stigmatising experiences and when they did engage with healthcare, they received poor quality care due to stigma and discrimination
[54]	UK	Migrants	Mental Health	12	Semi-structured interviews. Thematic analysis following Burnards method	Stigma prevented Syrian migrants from accessing mental health services because of fear of judgement from their communities
[55]	France, The Netherlands, Germany, and Greece	People who use drugs	N/A	99	Interviews. Qualitative content analysis	Stigma led to care avoidance and caused feelings of hopelessness
[56]	Germany	Migrants	Mental Health	20	Focus groups. Content structuring content analysis	Stigma prevented Syrian migrants from accessing mental health services because of fear of being labelled as "crazy" or becoming a source of gossip in their communities.
[57]	USA	People who use drugs	N/A	11	Focus groups. Framework analysis	People avoided preventative care providers for fear of judgement of their status as a drug user. When they did choose to engage with healthcare providers, they withheld information surrounding their drug use. Shame associated with drug use justified further drug use
[58]	USA	Sex workers	N/A	21	Interviews. Rapid content analysis	Participants withheld their status as a sex worker from healthcare providers to avoid stigmatising experiences. When they did disclose their status, they felt shamed and condescended to, which prevented future care access
[59]	Canada	People who use drugs	N/A	15	Interviews. Analytic methods not specified	Fear of being identified as a drug user by their communities resulted in refusal to engage with recovery services and hesitancy to contact emergency services during a drug use related emergency (eg. Overdose)
[60]	USA	Migrants	Hepatitis-B Infection	17	Interviews. Thematic analysis	Stigma surrounding Hepatitis-B prevented African migrants from accessing Hep-B testing and treatment services

^*^Overlapping stigma refers to secondary sources of stigma, beyond inclusion health status, that impacted health

### Stigma as a barrier to healthcare

The most common theme among the included studies was stigma as a barrier to healthcare. Participants in the included studies consistently reported that stigma posed a significant barrier to accessing healthcare. Many individuals avoided preventive care due to previous negative experiences and concerns about being stigmatised by providers on the basis of a health condition that they lived with (e.g. HIV/AIDS, Hepatitis) or behaviours they engaged in (e.g. drug use) [51, 54, 56, 60]. Avoidance behaviours extended to emergency care, with participants describing how past experiences of discrimination from healthcare providers made them hesitant to seek treatment, leading them to delay or refuse medical attention until their conditions became severe [43, 44]. Several studies highlighted that some participants actively concealed their use of drugs or participation in sex-work from providers due to fear of stigma, which ultimately limited their access to appropriate treatment and harm reduction services [41, 42, 47, 58].

*“I think usually like when I’m seeing like a primary care physician or something. I’ll kind of test the waters. Or I’ll give them, you know, half of it […] Because usually, I feel like it’s just I think that we face so much stigma and you would want to hope and be in good hopes that, you know, a health care provider would treat you without bias […] so sometimes I feel like it’s I’m better off by just not disclosing it.” – * [58]*. Pg. 5)*

Beyond interactions with healthcare providers, the anticipation of public stigma shaped participants' decisions regarding healthcare engagement. People in the included studies avoided harm reduction services [39, 48, 59], mental health treatment [38, 54, 56], and testing for bloodborne illnesses such as Hepatitis B or C and HIV [33, 46, 60] due to concerns about how they would be perceived by members of the public. For mothers with a history of substance use specifically, previous experiences of stigma led to social withdrawal and reluctance to participate in activities with their children, further reinforcing isolation [50].

Stigma surrounding medication for opioid use disorder (MOUD) was a common subtheme specific to people who used drugs. Participants reported that fear of being identified as a MOUD patient, either through social exposure or by having their status recorded in their medical history, deterred them from seeking treatment [39, 47, 51]. Some participants explicitly declined MOUD due to concerns about being labelled as having opioid use disorder [51]. The stigma attached to methadone treatment was particularly pronounced, with participants citing the visibility of daily clinic visits as a source of anxiety and a deterrent to engagement [51]. Additionally, family disapproval was identified as a barrier, as unfavourable attitudes toward opioid agonist therapy among relatives discouraged participants from seeking or continuing MOUD treatment [47].

*“My family is just not in my life right now. Even when I was not using and taking other medications, they would tell me that it was still a narcotic. They're pretty nasty with me. They think they know it all because they're older than me. While they drink every day, it's not okay for me to take medication every day. It makes no sense.” – * [47]*. Pg. 6)*

The cumulative effect of stigma in healthcare settings led to a pervasive distrust of medical professionals among participants in the studies. Many described feeling judged, ignored, or victimised when seeking care, which in turn fostered apprehension about engaging with the healthcare system in the future [33, 40, 45, 52, 58]. For some, this erosion of trust resulted in participants relying on emergency departments rather than preventive care, ultimately worsening health outcomes and placing additional strain on medical services [36, 57]; for others, care avoidance resulted in feelings of low-mood, depression, and anxiety [33, 58].

### Poor treatment as a result of stigma

Participants in the included studies frequently reported experiencing discrimination and inequitable treatment in healthcare settings due to their identities. Many people who used drugs described how clinicians' awareness of their drug use status negatively impacted the quality and timeliness of their care, particularly in emergency departments and hospital admissions [36, 42, 43, 47, 51, 53]; this theme was also present in the narratives of Aboriginal people in Canada, who felt narratives surrounding “drug-seeking natives” led providers to withhold pain medications and trivialise their health concerns [45] and among people engaged in sex-work [58]. Some participants perceived that their identities caused providers to deprioritise their care, resulting in long wait times, delayed treatment, and a general lack of attentiveness to their medical needs. This perception of being a lower priority than other patients contributed to feelings of frustration, a perception that their needs were not valued, anger, and distrust toward the healthcare system [43, 45, 47, 51, 58].

*“So many negative messages. Some are verbal. Some are nonverbal. I saw the nurse running around making sure everyone was warm, …you're giving other people blankets…. because I'm there… having a crisis…you're not going to treat someone kind, with humanity?” – * [51]*. Pg. 704)*

Several studies highlighted that participants felt that their identities influenced clinicians' prescribing practices, particularly regarding pain management. Many participants believed they were provided with insufficient pain relief due to physician assumptions about drug-seeking behaviour, which led to poor pain management, increased distress, and, in some cases, leaving the hospital against medical advice and future healthcare avoidance [36, 37, 41, 45, 51]. Participants with co-occurring health conditions, such as chronic pain or blood disorders, felt that their legitimate medical needs were often dismissed or downplayed because of their identities [37, 41, 51].

*[Healthcare providers] treated me like crap and I know it was because I was Native. We all know because of the look - there's a look. When you need the medical care we put up with it. We shouldn't have to. We bleed the same way, we birth the same way. We have no choice. Could be like [participant name], hasn't been to a doctor in 25 years. Can't all do that. – * [45]*. Pg. 89)*

Amongst people who use drugs specifically, stigma associated with substance use was also linked to dehumanisation and exclusion from adequate medical care. Participants described feeling ignored, shunned, or dismissed by healthcare providers, with some reporting that they were spoken to in a patronising manner or subjected to judgmental language that diminished their self-worth and dignity [42, 43, 52, 53]. In extreme cases, participants recounted instances in which healthcare staff explicitly devalued their lives, such as a nurse stating, *“Well, we're not going to waste a real bed on this person”* [51]. Pg. 704). Additionally, individuals who used injection drugs reported feeling particularly targeted by judgmental attitudes, citing instances where they were treated with disdain by hospital staff based on their status as injecting drug users [52, 53]. Such experiences contributed to a broader perception that addiction was not regarded as a legitimate illness, leading to further stigmatisation and reluctance to seek medical care [42, 51, 52].

### Stigma motivates continued drug use

Participants who used drugs in the included studies reported that internalised stigma related to substance use played a significant role in shaping their experiences with addiction and recovery. Internalised substance use stigma was more prevalent among participants than other forms of internalised stigma (eg. surrounding HIV or Heptatis-B/C Infection, surrounding their drug use), with many describing it as a contributing factor to their substance use [34, 57]. The internalisation of negative societal attitudes reinforced feelings of shame, low self-worth, and hopelessness, which, in turn, perpetuated continued drug use as a coping mechanism [49, 52, 57]. A participant in one study described the cyclical nature of substance abuse stigma.

*“I think what happened to me when I started using was the reason I’ve plummeted more into it… I was trying to numb every aspect of my emotions… I was trying to kill anything that felt vulnerable, like emotions. And I went from just puffs to at one point using the needles.” – * [34]*. Pg. 4)*

Some participants recounted how stigmatising interactions and external assumptions about their recovery process motivated them to continue using substances [35, 49]. Negative perceptions from others were internalised to the extent that individuals began to see themselves as incapable of change or improvement. Several participants described how being viewed as "hopeless losers" or as people who "could never amount to anything" reinforced a belief that they were inherently unworthy or incapable of leading a different life [49]. This pervasive sense of worthlessness created additional barriers to pursuing recovery, as participants questioned whether attempting to change was even worthwhile [49].

## Discussion

The findings of this review indicate that while stigma is widely acknowledged as a barrier to healthcare access for some inclusion health groups, particularly people who use drugs, the qualitative literature of how individuals experience stigma and perceive its direct impact on their health and wellbeing remains underdeveloped. Many of the included studies focused on how stigma influences healthcare-seeking behaviours, demonstrating that members of inclusion health groups often delay or avoid accessing care due to fear of discrimination and judgement, negative past experiences, or internalised shame. Although it is reasonable to infer that these behaviours contribute to worse health outcomes [61, 62], few studies explicitly examined the perceived long-term effects of stigma on physical health. This gap in the evidence suggests a need for research that moves beyond documenting healthcare avoidance and instead examines how people experience stigma-driven barriers and their transition into specific health consequences over time.

Despite the limited qualitative evidence linking stigma to physical health outcomes, the included studies provide clearer insights into its impact on mental health. Participants described feelings of low mood, depression, and anxiety in response to stigmatising encounters, indicating that stigma itself is a significant psychosocial stressor. Repeated exposure to stigma may contribute to chronic stress, social isolation, and diminished self-esteem [63–65], all of which are well documented risk factors for poor mental health [66–68]. Beyond these psychosocial effects, quantitative research has shown that chronic exposure to stigma can also contribute to physiological dysregulation, such as heightened stress response and increased allostatic load, which may help explain longer-term physical health impacts [69, 70]. These findings suggest that while stigma’s role in physical health remains under explored qualitatively, its detrimental effects on mental wellbeing are more evident and warrant further attention in both research and intervention efforts.

The vast majority of studies included in this review focused on people who use drugs, with significantly less attention given to other inclusion health groups such as sex workers and migrants, and none at all given to people in contact with the criminal justice system, people in indentured servitude or modern slavery, or members of Gypsy, Roma, and Traveller communities. This imbalance highlights a gap in the inclusion health literature, where the healthcare experiences of some marginalised populations are well documented, and others remain underexplored. The lack of research on inclusion health groups other than people who use drugs limits a comprehensive understanding of how intersecting vulnerabilities and stigma shape health. Expanding the focus of the inclusion health literature beyond people who use drugs is crucial for developing inclusive, evidence-based policies that improve healthcare access and outcomes for all inclusion health groups.

### Implications for the conceptualisation of stigma

The overrepresentation of studies on people who use drugs not only reflects an imbalance in the inclusion health literature but also highlights a deeper issue with how stigma is conceptualised in health research. As the results of this review demonstrate, much of the existing work on stigma tends to document its presence and immediate consequences, such as healthcare avoidance, discrimination, and psychological distress, without critically engaging with the broader structural forces that sustain and reinforce stigma. There is strong sociological evidence that stigma does not exist in isolation; it is embedded within social, economic, and political systems that marginalise certain groups while privileging others [13, 14, 71–73]. Despite the existing evidence, virtually all of the included studies are limited to describing individual experiences of stigma and fail to interrogate the structural forces, such as criminalisation, poverty and institutional discrimination that create and perpetuate these stigmatising conditions [74]. This results in a relatively superficial understanding of stigma’s role in perpetuating the health inequalities experienced by inclusion health groups, where the focus is on its effects rather than its root causes.

This limitation is particularly problematic given that inclusion health groups face intersecting forms of marginalisation that go beyond individual discrimination. For example, the stigma experienced by migrants is shaped by immigration policies and legal restrictions that limit access to healthcare [75, 76], while sex workers and people who use drugs face criminalisation and social exclusion that place them at increased risk of violence and poor health outcomes [77, 78]. Similarly, Indigenous populations often experience stigma within the context of colonial histories, systemic racism, and ongoing socioeconomic disadvantage [79, 80]. Without acknowledging these structural determinants, research on the impacts of stigma runs the risk of reinforcing an individualised narrative that places the burden of stigma on those experiencing it, rather than on the structural forces that produce and sustain stigma as a mechanism of exclusion; this is a form of symbolic violence [71] and is an important factor perpetuating health inequalities [29]. Expanding stigma research to take a more structural approach would provide a more complete understanding of its impact on health and highlight pathways for policy interventions that go beyond individual level stigma reduction and target the structural forces that create and maintain health inequalities.

This critique of the literature is similar to past calls from Hatzenbuehler and colleagues to address the structural and social-determinant dimensions of stigma, recognising that many of its health effects are produced and sustained through upstream systems such as policy, law, and institutional practice [22, 81]. This perspective aligns with Fraser et al. [82], who conceptualise addiction stigma through the lens of stigma power and the biopolitics of liberal modernity, showing how stigma operates as a political technology that governs and disciplines marginalised populations. Similarly, Magno et al. [83] highlight how stigma is embedded within broader political and economic relations, calling for greater attention to processes of resistance as well as domination. While this body of theoretical work offers valuable guidance, the findings of this review indicate that there remains a gap between these conceptual advances and their application within public health research. Researchers employing the concept of stigma in applied health contexts need to engage more directly with its structural and political dimensions, ensuring that interventions target not only attitudes and behaviours but also the systems that reproduce exclusion and inequality.

### Rethinking inclusion health

While the concept of inclusion health groups is useful in drawing attention to populations that experience extreme health inequalities, it also raises questions about how we categorise and address marginalisation in public health research. By grouping together people who use drugs, migrants, sex workers, Indigenous populations, homeless people, people in contact with the justice system, members of Gypsy, Roma, and Traveller communities, and others under a single umbrella, the inclusion health framework risks treating these groups as inherently separate from the broader population rather than as individuals shaped by shared structural forces. This segmentation may inadvertently obscure the root causes of health inequity, such as austerity policies [84], punitive legal systems [85], racial capitalism [86], and entrenched poverty [87], by focusing on their status as "vulnerable groups" rather than on the systems that actively produce their vulnerability [13]. The obscurement of the structural drivers of health inequalities is an important force in maintaining and reinforcing their existence [29].

A more productive approach would shift the emphasis from categorising marginalised populations towards addressing the upstream drivers of their exclusion. Rather than centring research on how stigma impacts inclusion health groups as isolated entities, greater attention should be paid to the political and economic structures that create and sustain their marginalisation. Criminalisation, for example, is not just a consequence of stigma, but also a deliberate mechanism of social control that disproportionately affects people who use drugs, sex workers, and migrants [88–90]. If public health research continues to focus primarily on examining the downstream effects of stigma, rather than the systemic structures that create it, we risk perpetuating a cycle in which inclusion health groups remain on the margins without addressing why these margins exist in the first place.

This critique aligns with longstanding debates in the health inequalities literature, which emphasise the need to focus on the structural determinants of health rather than solely on the outcomes experienced by marginalised groups. Scholars studying health inequalities such as Clare Bambra [91, 92] and Michael Marmot [7, 93], amongst others [94, 95], argue that health inequalities are not the result of individual behaviours or group specific vulnerabilities, but are instead produced by broader socioeconomic and political structures. The inclusion health framework, while useful for highlighting the extreme disadvantages faced by certain populations, risks reinforcing a deficit-based approach that focuses on the characteristics of marginalised groups rather than interrogating the systems that produce their marginalisation. Wacquant’s [96] work on advanced marginality and territorial stigma further challenges the notion that inclusion health groups should be treated as distinct categories. His research demonstrates how poverty, criminalisation, and social exclusion are not individual or group specific experiences, but are instead embedded in spatial and economic processes that systematically disadvantage certain populations [96, 97]. Similarly, Scambler [17] argues that stigma is not an isolated phenomenon affecting particular groups, but a function of broader systems of neoliberal power that serve to justify and maintain social inequities. The findings of this review reinforce these existing critiques of deficit-based approaches to health inequalities and highlight the need for research that prioritises structural determinants over group-based categorisations. Rather than focusing on inclusion health groups as isolated entities, future research should align with broader scholarship on social determinants of health, emphasising the systemic forces that drive exclusion and poor health outcomes across marginalised populations.

### Strengths and limitations

To our knowledge, this is the first review to examine the impact of stigma on the health and wellbeing of inclusion health groups, providing a novel synthesis of qualitative evidence on this topic. By mapping the existing literature, this review highlights important gaps and imbalances in how the effects of stigma on health is studied across different populations, particularly the overrepresentation of research on people who use drugs relative to other inclusion health groups. This review was conducted in line with accepted guidance on scoping review design and reporting [30]. In doing so, it provides a foundation for future research, emphasising the need for more structurally oriented approaches that move beyond documenting individual experiences of stigma to interrogating the broader systems that sustain health inequalities.

This review has several limitations. Despite following accepted guidance on scoping review design and reporting, due to its nature as a scoping review some relevant studies may have been missed that could have been identified through the more rigorous search and screening processes of a full systematic review. Additionally, the decision to exclude quantitative research limits our ability to assess the measurable impact of stigma on health outcomes, such as morbidity and mortality rates, which would provide a more comprehensive understanding of its effects. Another key limitation is the absence of a formal quality appraisal of included studies, which means that variations in methodological rigour across studies were not systematically assessed. As a result, the strength of the evidence base discussed here remains uncertain. Most of the included literature was conducted in North America. It is possible that the existing literature from other geographic settings is more comprehensive or operationalises stigma differently than the literature included here. Additionally, our exclusion criteria excluded studies not published in English; it is possible that the inclusion of non-English literature could have provided greater insight into the impacts of stigma on health. This review focused on adult populations (aged 18 and over), as the determinants and manifestations of stigma, and their health impacts, differ for children and adolescents. This exclusion represents a limitation, as understanding stigma among younger populations is also critical; future reviews should specifically examine how stigma affects the health and wellbeing of under-18 s within inclusion health groups. Finally, by relying on existing literature, this review is constrained by the limitations of the field itself, including the narrow focus on substance use and the lack of engagement with structural determinants of stigma, which reinforces the need for future research that expands beyond these confines.

## Conclusion

The findings of this review suggest that inclusion health groups primarily experience stigma as a barrier to accessing healthcare. Across the studies, participants reported avoiding medical services due to fear of discrimination, previous negative experiences with healthcare providers, and concerns about being labelled or judged. This avoidance behaviour was particularly evident among people who use drugs, who frequently described reluctance to seek treatment for fear of being dismissed, mistreated, or denied appropriate care. While these experiences were well-documented in the literature on drug use, there was comparatively little research on how other inclusion health groups, such as migrants, sex workers, and Indigenous populations, experience stigma leaving significant gaps surrounding how stigma impacts health across different inclusion health groups. The qualitative evidence linking stigma to health outcomes was sparse, as most studies focused on its role in shaping healthcare use behaviours rather than long term health consequences. Examining stigma’s impact across multiple populations, rather than in isolation, is crucial for identifying commonalities in experience that point to structural rather than solely individual level causes. A broader evidence base, that cuts across populations, would help to better understand the structural nature of stigma and inform more comprehensive, structural interventions.

 Inclusion health as a framework brings attention to populations facing extreme health inequalities, but its reliance on group-based categorisation risks obscuring the broader structural forces that drive health inequalities and thus perpetuate their existence. This review highlights how, in the context of inclusion health groups, stigma research often focuses on individual experiences of discrimination while neglecting the structural forces that produce and sustain social inequity. Without shifting focus towards these upstream factors, there is a risk of reinforcing deficit-based narratives that health inequalities stem from within group behaviours rather than from harmful social structures. Future research should move beyond documenting stigma’s effects on individuals towards interrogating the systemic conditions that generate stigma and perpetuate health inequalities.

## Supplementary Information

Supplementary Material 1.

## Data Availability

The datasets used and/or analysed during the current study are available from the corresponding author on reasonable request.

## References

[1] Case A, Kraftman L. Health inequalities. Oxford Open Economics. 2024;3:i499–528.

[2] Goldblatt P. Health inequalities, lives cut short. London: UCL Institute of Health Equity; 2024.

[3] Marmot M, Allen J, Boyce TG, P & Morisson J.Health equity in England: the Marmot review 10 years on [Online]. The Health Foundation. 2020. Available: https://www.health.org.uk/publications/reports/the-marmot-review-10-years-on. Accessed 15/03/2022.

[4] Mezzina R, Gopikumar V, Jenkins J, Saraceno B, Sashidharan S. Social vulnerability and mental health inequalities in the “Syndemic”: Call for action. Front Psychiatry. 2022;13:894370. 35747101 10.3389/fpsyt.2022.894370 PMC9210067

[5] Schneider SM, Roots A, Rathmann K. Health outcomes and health inequalities. 2021.

[6] Bambra C, Riordan R, Ford J, Matthews F. The COVID-19 pandemic and health inequalities. J Epidemiol Community Health. 2020;74:964–8. 32535550 10.1136/jech-2020-214401 PMC7298201

[7] Marmot M. Health equity in England: the Marmot review 10 years on. BMJ. 2020. 10.1136/bmj.m693. 32094110 10.1136/bmj.m693

[8] Meadows J, Montano M, Alfar AJK, Başkan ÖY, De Brún C, Hill J, et al. The impact of the cost-of-living crisis on population health in the UK: rapid evidence review. BMC Public Health. 2024;24:561. 38388342 10.1186/s12889-024-17940-0 PMC10882727

[9] England N. Inclusion Health Groups [Online]. 2021. Available: https://www.england.nhs.uk/about/equality/equality-hub/national-healthcare-inequalities-improvement-programme/what-are-healthcare-inequalities/inclusion-health-groups/ Accessed 02/04/2025.

[10] Bramley G, Fitzpatrick S, Sosenko F. Mapping the “hard edges” of disadvantage in England: Adults involved in homelessness, substance misuse, and offending. Geogr J. 2020;186:390–402.

[11] Tyler I, Slater T. Rethinking the sociology of stigma. Sociol Rev. 2018. 10.1177/0038026118777425.

[12] Tweed EJ, Popham F, Thomson H, Katikireddi SV. Including ‘inclusion health’? A discourse analysis of health inequalities policy reviews. Crit Public Health. 2022;32:700–12.

[13] Addison M, Scott S, Bambra C, Lhussier M. Stigma and the inverse care law: experiences of ‘Care’for People Living in Marginalised conditions. Sociol Health Illn. 2025;47:e70000. 39829101 10.1111/1467-9566.70000 PMC11744054

[14] Addison M, Lhussier M, Bambra C. Relational stigma as a social determinant of health: “I’m not what you _____see me as.” SSM - Qualitative Research in Health. 2023;4:100295.

[15] Mitchell UA, Nishida A, Fletcher FE, Molina Y. The long arm of oppression: how structural stigma against marginalized communities perpetuates within-group health disparities. Health Educ Behav. 2021;48:342–51. 34080480 10.1177/10901981211011927

[16] Hicks S, Lewis C. Nobody becomes stigmatised ‘all at once’: An interactionist account of stigma on a modernist council estate. Sociol Rev. 2020;68:1370–85.

[17] Scambler G. Heaping blame on shame: ‘Weaponising stigma’ for neoliberal times. Sociol Rev. 2018;66:766–82.

[18] Tyler I. Stigma : the machinery of inequality. London: Zed Books Ltd; 2020.

[19] Scambler G. Health-related stigma. Soc Health Illn. 2009;31:441–55. 10.1111/j.1467-9566.2009.01161.x 19366430

[20] Link BG, Phelan JC. Conceptualizing stigma. Annu Rev Sociol. 2001;27:363–85.

[21] Link BG, Phelan J. Stigma power. Soc Sci Med. 2014;103:24–32. 24507908 10.1016/j.socscimed.2013.07.035 PMC4451051

[22] Hatzenbuehler ML, Phelan JC, Link BG. Stigma as a fundamental cause of population health inequalities. Am J Public Health. 2013;103:813–21. 23488505 10.2105/AJPH.2012.301069 PMC3682466

[23] Yang LH, Wong LY, Grivel MM, Hasin DS. Stigma and substance use disorders: an international phenomenon. Curr Opin Psychiatry. 2017;30:378–88. 28700360 10.1097/YCO.0000000000000351 PMC5854406

[24] Cabieses B, Belo K, Calderón AC, Rada I, Rojas K, Araoz C, et al. The impact of stigma and discrimination-based narratives in the health of migrants in Latin America and the Caribbean: a scoping review. Lancet Regional Health – Americas. 2024. 10.1016/j.lana.2023.100660. 39763497 10.1016/j.lana.2023.100660 PMC11703579

[25] Douglass CH, Win TM, Goutzamanis S, Lim MSC, Block K, Onsando G, et al. Stigma associated with alcohol and other drug use among people from migrant and ethnic minority groups: results from a systematic review of qualitative studies. J Immigr Minor Health. 2023;25:1402–25. 36976449 10.1007/s10903-023-01468-3 PMC10632266

[26] Martin KD, Taylor A, Howell B, Fox AD. Does criminal justice stigma affect health and healthcare utilization?: A systematic review of public health and medical literature. Int J Prison Health. 2020;16:263–79. 33634660 10.1108/IJPH-01-2020-0005 PMC11016312

[27] Arksey H, O’Malley L. Scoping studies: towards a methodological framework. Int J Soc Res Methodol. 2005;8:19–32.

[28] PRICE, T. 2025. Exploring the impact of stigma on the health of those who can be defined as belonging to inclusion health groups – protocol for a scoping review of qualitative studies [Online]. Open Science Framework. Available: 10.17605/OSF.IO/4VGDH Accessed 02/04/2025.

[29] Price TJ, Mcgowan VJ. A cycle of social violence: a novel theoretical framework for explaining how structural, slow, and symbolic violence interact to produce and maintain health inequalities in England. Soc Sci Med. 2025;383:118438. 40695055 10.1016/j.socscimed.2025.118438 PMC7619319

[30] Tricco AC, Lillie E, Zarin W, O’Brien KK, Colquhoun H, Levac D, et al. PRISMA extension for scoping reviews (PRISMA-ScR): checklist and explanation. Ann Intern Med. 2018;169:467–73. 30178033 10.7326/M18-0850

[31] England N. A national framework for NHS – action on inclusion health [Online]. NHS England. 2023. Available: https://www.england.nhs.uk/long-read/a-national-framework-for-nhs-action-on-inclusion-health/ Accessed 26/03/2025.

[32] Peters MD, Godfrey CM, Khalil H, Mcinerney P, Parker D, Soares CB. Guidance for conducting systematic scoping reviews. Int J Evid Based Healthc. 2015;13:141–6. 26134548 10.1097/XEB.0000000000000050

[33] Arrey AE, Bilsen J, Lacor P, Deschepper R. Perceptions of stigma and discrimination in health care settings towards sub-Saharan African migrant women living with HIV/AIDS in Belgium: a qualitative study. J Biosoc Sci. 2017;49:578–96. 27692006 10.1017/S0021932016000468

[34] Batchelder AW, Foley JD, Kim J, Thiim A, Kelly J, Mayer K, et al. Intersecting internalized stigmas and HIV self-care among men who have sex with men and who use substances. Soc Sci Med. 2021;275:113824. 33721745 10.1016/j.socscimed.2021.113824 PMC8009855

[35] Burgess A, Bauer E, Gallagher S, Karstens B, Lavoie L, Ahrens K, et al. Experiences of stigma among individuals in recovery from opioid use disorder in a rural setting: a qualitative analysis. J Subst Abuse Treat. 2021;130:108488. 34118715 10.1016/j.jsat.2021.108488

[36] Chan Carusone S, Guta A, Robinson S, Tan DH, Cooper C, O’Leary B, et al. Maybe if i stop the drugs, then maybe they’d care?”—hospital care experiences of people who use drugs. Harm Reduct J. 2019;16:1–10. 30760261 10.1186/s12954-019-0285-7 PMC6373073

[37] Collins AB, Baird J, Nimaja E, Ashenafi Y, Clark MA, Beaudoin FL. Experiences of patients at high risk of opioid overdose accessing emergency department and behavioral health interventions: a qualitative analysis in an urban emergency department. BMC Health Serv Res. 2023;23:370. 37069593 10.1186/s12913-023-09387-7 PMC10110343

[38] Davy B, Riosa PB, Ghassemi E. Understanding the mental health perspectives and experiences of migrants to Canada. Int J Health Serv. 2024;54:52–64. 10.1177/27551938231156032 36798040

[39] Fiddian-Green A, Gubrium A, Harrington C, Evans EA. Women-reported barriers and facilitators of continued engagement with medications for opioid use disorder. Int J Environ Res Public Health. 2022;19:9346. 35954700 10.3390/ijerph19159346 PMC9368271

[40] Fontaine G, Presseau J, Bruneau J, Etherington C, Thomas IM, Hung J-HC, et al. Using an intersectionality lens to explore barriers and enablers to hepatitis C point-of-care testing: a qualitative study among people who inject drugs and service providers. Int J Equity Health. 2024;23:124. 38886803 10.1186/s12939-024-02209-0 PMC11184812

[41] Forchuk C, Serrato J, Scott L. People with lived and living experience of methamphetamine use and admission to hospital: what harm reduction do they suggest needs to be addressed? Health Promot Chronic Dis Prev Can. 2023;43:338. 37466399 10.24095/hpcdp.43.7.04 PMC10414816

[42] Forchuk C, Serrato J, Scott L. Perceptions of stigma among people with lived experience of methamphetamine use within the hospital setting: qualitative point-in-time interviews and thematic analyses of experiences. Front Public Health. 2024;12:1279477. 38414902 10.3389/fpubh.2024.1279477 PMC10896942

[43] Galarneau LR, Scheuermeyer FX, Hilburt J, O’Neill ZR, Barbic S, Moe J, et al. Qualitative exploration of emergency department care experiences among people with opioid use disorder. Ann Emerg Med. 2023;82:1–10. 36967276 10.1016/j.annemergmed.2023.02.007 PMC12129082

[44] Garcia V, McCann L, Lauber E, Vaccaro C, Swauger M, Heckert DA. Opioid overdoses and take-home naloxone interventions: ethnographic evidence for individual-level barriers to treatment of opioid use disorders in rural Appalachia. Subst Use Misuse. 2024;59:1313–22. 38635977 10.1080/10826084.2024.2340986

[45] Goodman A, Fleming K, Markwick N, Morrison T, Lagimodiere L, Kerr T, et al. “They treated me like crap and I know it was because I was Native”: The healthcare experiences of Aboriginal peoples living in Vancouver’s inner city. Soc Sci Med. 2017;178:87–94. 28214449 10.1016/j.socscimed.2017.01.053 PMC5367883

[46] Goodyear T, Brown H, Browne AJ, Hoong P, Ti L, Knight R. Stigma is where the harm comes from”: exploring expectations and lived experiences of hepatitis C virus post-treatment trajectories among people who inject drugs. Int J Drug Policy. 2021;96:103238. 33902968 10.1016/j.drugpo.2021.103238 PMC8881088

[47] Hsu M, Jung OS, Kwan LT, Jegede O, Martin B, Malhotra A, et al. Access challenges to opioid use disorder treatment among individuals experiencing homelessness: voices from the streets. J Subst Use Addict Treat. 2024;157:209216. 37981243 10.1016/j.josat.2023.209216

[48] Ibragimov U, Cooper KE, Batty E, Ballard AM, Fadanelli M, Gross SB, et al. Factors that influence enrollment in syringe services programs in rural areas: a qualitative study among program clients in Appalachian Kentucky. Harm Reduct J. 2021;18:68. 34193165 10.1186/s12954-021-00518-z PMC8244225

[49] Judd H, Yaugher AC, O’Shay S, Meier CL. Understanding stigma through the lived experiences of people with opioid use disorder. Drug Alcohol Depend. 2023;249:110873. 37390780 10.1016/j.drugalcdep.2023.110873

[50] Lochhead L, Addison M, Cavener J, Scott S, Mcgovern W. Exploring the impact of stigma on health and wellbeing: insights from mothers with lived experience accessing recovery services. Int J Environ Res Public Health. 2024;21:1189. 39338072 10.3390/ijerph21091189 PMC11430851

[51] McCurry MK, Avery-Desmarais S, Schuler M, Tyo M, Viveiros J, Kauranen B. Perceived stigma, barriers, and facilitators experienced by members of the opioid use disorder community when seeking healthcare. J Nurs Scholarsh. 2023;55:701–10. 36317787 10.1111/jnu.12837

[52] Muncan B, Walters SM, Ezell J, Ompad DC. They look at us like junkies”: influences of drug use stigma on the healthcare engagement of people who inject drugs in New York City. Harm Reduct J. 2020;17:1–9. 32736624 10.1186/s12954-020-00399-8 PMC7393740

[53] Paquette CE, Syvertsen JL, Pollini RA. Stigma at every turn: health services experiences among people who inject drugs. Int J Drug Policy. 2018;57:104–10. 29715589 10.1016/j.drugpo.2018.04.004 PMC5994194

[54] Paudyal P, Tattan M, Cooper MJ. Qualitative study on mental health and well-being of Syrian refugees and their coping mechanisms towards integration in the UK. BMJ Open. 2021;11:e046065. 34417211 10.1136/bmjopen-2020-046065 PMC8381320

[55] Pouille A, De Ruysscher C, van Selm L, van Amsterdam J, van den Brink W, Busz M, Perez Gayo R, Atzemis M, Vanderplasschen W, & ELISABETH, S.-E. C. M. J. B. I. P. R. B. S. K. R. L. A. L. M. D. V. A. 2024. Challenges and support needs among persons with a migration background who use drugs in four European metropolitan cities. Harm Reduct J. 21;208. 10.1186/s12954-024-01110-x PMC11585216 39580438

[56] Renner A, Hoffmann R, Nagl M, Roehr S, Jung F, Grochtdreis T, et al. Syrian refugees in Germany: perspectives on mental health and coping strategies. J Psychosom Res. 2020;129:109906. 31884301 10.1016/j.jpsychores.2019.109906

[57] Schuler MS, Seney V. “It’s My Secret”: shame as a barrier to care in individuals with opioid use disorder. J Am Psychiatr Nurses Assoc. 2024;30:456–64. 38581184 10.1177/10783903241242748

[58] Singer RB, Johnson AK, Crooks N, Bruce D, Wesp L, Karczmar A, et al. Feeling safe, feeling seen, feeling free”: combating stigma and creating culturally safe care for sex workers in Chicago. PLoS ONE. 2021;16:e0253749. 34185795 10.1371/journal.pone.0253749 PMC8241054

[59] Viste D, Rioux W, Medwid M, Williams K, Tailfeathers E, Lee A, et al. Perceptions of overdose response hotlines and applications among rural and remote individuals who use drugs in Canada: a qualitative study. Health Promot Chronic Dis Prev Can. 2024;44:471. 39607434 10.24095/hpcdp.44.11/12.03 PMC11728862

[60] Wang M, Qureshi A, Johnson N, Mansalay A, Muhr A, Abatemarco DJ, et al. A health belief model examination of factors related to hepatitis B screening among African immigrants in Philadelphia. J Racial Ethn Health Disparities. 2024;11:3907–16. 37878235 10.1007/s40615-023-01841-w

[61] Cuschieri S, Mamo J. Taking care of the ordinary in extraordinary times—delayed routine care means more morbidity and pre-mature mortality. Eur J Public Health. 2021;31:iv27–30. 34751363 10.1093/eurpub/ckab156 PMC8576302

[62] Papalamprakopoulou Z, Ntagianta E, Triantafyllou V, Kalamitsis G, Dharia A, Dickerson SS, et al. Breaking the vicious cycle of delayed healthcare seeking for people who use drugs. Harm Reduct J. 2025;22:1–10. 40045378 10.1186/s12954-025-01166-3 PMC11881266

[63] Crocker J, Major B. Social stigma and self-esteem: the self-protective properties of stigma. Psychol Rev. 1989;96:608.

[64] Link BG, Phelan JC. Stigma and its public health implications. Lancet. 2006;367:528–9. 16473129 10.1016/S0140-6736(06)68184-1

[65] Major B, Eccleston CP. Stigma and social exclusion. Social psychology of inclusion and exclusion: Psychology Press; 2004.

[66] Orth U, Robins RW. Understanding the link between low self-esteem and depression. Curr Dir Psychol Sci. 2013;22:455–60.

[67] Patel RS, Wardle K, Parikh RJ. Loneliness: the present and the future. Age Ageing. 2019;48:476–7. 30927406 10.1093/ageing/afz026

[68] Ross RA, Foster SL, Ionescu DF. The role of chronic stress in anxious depression. Chronic Stress. 2017;1:2470547016689472. 32440578 10.1177/2470547016689472 PMC7219927

[69] Juster R-P, Rutherford C, Keyes K, Hatzenbuehler ML. Associations between structural stigma and allostatic load among sexual minorities: results from a population-based study. Psychosom Med. 2024;86:157–68. 38345315 10.1097/PSY.0000000000001289

[70] Mijas M, Blukacz M, Koziara K, Kasparek K, Pliczko MP, Galbarczyk A, et al. Dysregulated by stigma: cortisol responses to repeated psychosocial stress in gay and heterosexual men. Psychoneuroendocrinology. 2021;131:105325. 34171795 10.1016/j.psyneuen.2021.105325

[71] Bourdieu P. Outline of a theory of practice. Cambridge: Cambridge University Press; 1977.

[72] Burchardt T, Le Grand J, Piachaud D, Hills J, Grand L. Understanding social exclusion. London: London School of Economics; 2002.

[73] Friedman SR, Williams LD, Guarino H, Mateu-Gelabert P, Krawczyk N, Hamilton L, et al. The stigma system: how sociopolitical domination, scapegoating, and stigma shape public health. J Community Psychol. 2022;50:385–408. 34115390 10.1002/jcop.22581 PMC8664901

[74] Kitching GT, Firestone M, Schei B, Wolfe S, Bourgeois C, O’Campo P, et al. Unmet health needs and discrimination by healthcare providers among an Indigenous population in Toronto, Canada. Can J Public Health. 2020;111:40–9. 31435849 10.17269/s41997-019-00242-z PMC7046890

[75] Larchanché S. Intangible obstacles: health implications of stigmatization, structural violence, and fear among undocumented immigrants in France. Soc Sci Med. 2012;74:858–63. 22000263 10.1016/j.socscimed.2011.08.016

[76] Lebrón AMW, Schulz AJ, Gamboa C, Reyes A, Viruell-Fuentes EA, Israel BA. “They Are Clipping Our Wings”: health implications of restrictive immigrant policies for Mexican-Origin women in a northern border community. Race Soc Probl. 2018;10:174–92.

[77] Cohen A, Vakharia SP, Netherland J, Frederique K. How the war on drugs impacts social determinants of health beyond the criminal legal system. Focus. 2024;22:515–26. 39563880 10.1176/appi.focus.24022021 PMC11571189

[78] Sanders T. Inevitably violent? Dynamics of space, governance, and stigma in understanding violence against sex workers. Special issue: Problematizing prostitution: critical research and scholarship. Emerald Group Publishing Limited. 2016.

[79] Robertson DL. Invisibility in the color-blind era: examining legitimized racism against Indigenous peoples. Am Indian Q. 2015;39:113–53.

[80] Smye V, Browne AJ, Josewski V, Keith B, Mussell W. Social suffering: Indigenous peoples’ experiences of accessing mental health and substance use services. Int J Environ Res Public Health. 2023;20:3288. 36833982 10.3390/ijerph20043288 PMC9958899

[81] Hatzenbuehler ML, Link BG. Introduction to the special issue on structural stigma and health. Soc Sci Med. 2014;103:1–6. 24445152 10.1016/j.socscimed.2013.12.017

[82] Fraser S, Pienaar K, Dilkes-Frayne E, Moore D, Kokanovic R, Treloar C, et al. Addiction stigma and the biopolitics of liberal modernity: a qualitative analysis. Int J Drug Policy. 2017;44:192–201. 28366599 10.1016/j.drugpo.2017.02.005

[83] MagnoTertoParker LVR. Stigmatisation and resistance processes: reflections on the field of HIV research and an agenda for contemporary stigma studies. Glob Public Health. 2024;19:2371390. 39016193 10.1080/17441692.2024.2371390

[84] Ginn J. Austerity and inequality. Exploring the impact of cuts in the UK by gender and age. Res Ageing Soc Policy. 2013;1:28–53.

[85] Massoglia M. Incarceration, health, and racial disparities in health. Law Soc Rev. 2008;42:275–306.

[86] Deangelis RT. Racial Capitalism and Black–White Health Inequities in the United States: The Case of the 2008 Financial Crisis. J Health Soc Behav 00221465241260103. 2024. 10.1177/00221465251338975 40405683

[87] Chokshi DA. Income, poverty, and health inequality. JAMA. 2018;319:1312–3. 29614168 10.1001/jama.2018.2521

[88] Dalesandry M. Persistent Criminalization as a Protracted Crisis: Stigma and Rational Choice Within the Sex Workers’ Rights Community. George Mason University. 2023.

[89] Henry S, Vandersip J, Anastasia DJ. Crime, Justice, and Social Control, Cognella, Incorporated. 2019.

[90] Scher BD, Neufeld SD, Butler A, Bonn M, Zakimi N, Farrell J, et al. “Criminalization causes the stigma”: perspectives from people who use drugs. Contemp Drug Probl. 2023;50:402–25.

[91] Bambra C. Health divides: Where you live can kill you, Bristol, Bristol: Policy Press. 2016.

[92] BBambra C. Health in Hard Times, Bristol, Bristol: Policy Press. 2019.

[93] Marmot M. Social determinants of health inequalities. Lancet. 2005;365:1099–104. 15781105 10.1016/S0140-6736(05)71146-6

[94] LYNCH, J. 2020. Regimes of inequality: the political economy of health and wealth, Cambridge University Press.

[95] Raphael D. The political economy of health: a research agenda for addressing health inequalities in Canada. Can Public Policy. 2015;41:S17–25.

[96] Wacquant L. Territorial stigmatization in the age of advanced marginality. Thesis Eleven. 2007;91:66–77.

[97] Wacquant L. Revisiting territories of relegation: class, ethnicity and state in the making of advanced marginality. Urban Stud. 2016;53:1077–88.

