# Supplementary material for: Exploring the impact of stigma on the health of inclusion health groups: a qualitative scoping review and critical analysis
Source: BMC Public Health. 2025 Nov 24;26:24. doi: 10.1186/s12889-025-25603-x (PMC12764128; doi:10.1186/s12889-025-25603-x)
Supplement: Supplementary file 1 — Supplementary Material 1. [file 12889_2025_25603_MOESM1_ESM.docx]

Supplementary Table 1: Search Strategy

| SPIDER concept | Search Terms |
| --- | --- |
| S- Sample: Inclusion health groups | inclusion health groups Homeless* OR unhoused* OR roofless* OR traveller* OR traveler* OR roma* or gyps* OR aboriginal* OR first nation* OR indigenous people* OR* OR sex worker* OR prostitu* OR addict* OR substance abuse* OR substance use* OR alcohol* OR refugee* OR asylum* OR forced migra* OR immigra* OR probation* OR criminal* OR incarcerat* OR felon* OR imprison* OR enslave*OR *indentur* OR traffic* OR inclusion health* |
| AND | |
| PI – Phenomenon(s) of interest: Stigma | Stigma* OR reputation* OR public opinion* OR prejudice* OR defam* OR depict* OR imag* OR label* OR portray* OR representation* OR rough OR sensational* OR stereotyp* OR taint* OR vilif* OR shame* |
| AND | |
| PI – Phenomenon(s) of interest: Health | health health* OR health condition* OR health behav* OR mental health* OR wellbeing* |
| AND | |
| D - Design: qualitative research methods | Qualitative OR qualitative analysis OR qualitative research OR participatory OR grounded theory OR ethnograph* OR phenomenolog* OR feminis* OR narrative* OR Interview* OR focus group* OR case stud* OR anthrop* OR thematic OR constant comparative OR observ* OR field notes |
| AND | |
| E - Evaluation: Experience | Perceive* OR perception* OR perspective* OR view* OR experienc* OR attitude* OR belief* OR opinion* OR feel* OR know* OR understand* OR thought* OR impress* OR stance* OR standpoint* OR position* |
| R – Research type: Qualitative | See ‘design’ |
